# Alpha-Fetoprotein Detection of Hepatocellular Carcinoma Leads to a Standardized Analysis of Dynamic AFP to Improve Screening Based Detection

**DOI:** 10.1371/journal.pone.0156801

**Published:** 2016-06-16

**Authors:** Thomas G. Bird, Polyxeni Dimitropoulou, Rebecca M. Turner, Sara J. Jenks, Pearce Cusack, Shiying Hey, Andrew Blunsum, Sarah Kelly, Catharine Sturgeon, Peter C. Hayes, Sheila M. Bird

**Affiliations:** 1 Centre for Liver and Digestive Disorders, Royal Infirmary of Edinburgh, Edinburgh, EH16 4SA, United Kingdom; 2 Cancer Research UK Beatson Institute, Glasgow, G61 1BD, United Kingdom; 3 MRC Biostatistics Unit, Cambridge, CB2 0SR, United Kingdom; 4 Department of Clinical Biochemistry, Royal Infirmary of Edinburgh, Edinburgh, EH16 4SA, United Kingdom; 5 Department of Mathematics and Statistics, Strathclyde University, Glasgow, G1 1XH, United Kingdom; Yonsei University College of Medicine, REPUBLIC OF KOREA

## Abstract

Detection of hepatocellular carcinoma (HCC) through screening can improve outcomes. However, HCC surveillance remains costly, cumbersome and suboptimal. We tested whether and how serum Alpha-Fetoprotein (AFP) should be used in HCC surveillance. Record linkage, dedicated pathways for management and AFP data-storage identified i) consecutive highly characterised cases of HCC diagnosed in 2009–14 and ii) a cohort of ongoing HCC-free patients undergoing regular HCC surveillance from 2009. These two well-defined Scottish patient cohorts enabled us to test the utility of AFP surveillance. Of 304 cases of HCC diagnosed over 6 years, 42% (129) were identified by a dedicated HCC surveillance programme. Of these 129, 47% (61) had a detectable lesion first identified by screening ultrasound (US) but 38% (49) were prompted by elevated AFP. Despite pre-HCC diagnosis AFP >20kU/L being associated with poor outcome, ‘AFP-detected’ tumours were offered potentially curative management as frequently as ‘US-detected’ HCCs; and had comparable survival. Linearity of serial log_10_-transformed AFPs in HCC cases and in the screening ‘HCC-free’ cohort (n = 1509) provided indicators of high-risk AFP behaviour in HCC cases. An algorithm was devised in static mode, then tested dynamically. A case/control series in hepatitis C related disease demonstrated highly significant detection (p<1.72*10^−5^) of patients at high risk of developing HCC. These data support the use of AFP in HCC surveillance. We show proof-of-principle that an automated and further refine-able algorithmic interpretation of AFP can identify patients at higher risk of HCC. This approach could provide a cost-effective, user-friendly and much needed addition to US surveillance.

## Introduction

Hepatocellular carcinoma (HCC) is an enormous burden to global health. In adult men HCC is now the fifth most frequently diagnosed cancer, and the second leading cause of cancer-related death worldwide [1, 2]. Despite widely implemented screening programmes and improved therapies, HCC is predicted to continue as a major problem for the foreseeable future. Late HCC detection, particularly in symptomatic patients, leaves few effective therapeutic options and is associated with extremely poor outcome [2, 3]. However, the early detection of asymptomatic HCC through screening programmes in high risk populations has improved outcomes in specific contexts and does permit potentially curative therapies [4–7].

Based upon the high risk of HCC in a large but definable population, combined with minimally invasive screening tests and genuine curative options for early cancers, HCC represents a suitable target for surveillance programmes. However, the optimal methodology for implementing HCC surveillance remains highly debated.

Serum Alpha-Fetoprotein (AFP) is the most widely used biomarker in HCC surveillance programmes and, until recently, was included in international guidelines for HCC surveillance [8–10]. AFP has significant limitations as a screening test: specifically, in one third to half of HCC cases AFP will not be significantly elevated [11–14]. Conversely, AFP can be chronically elevated or time-varying in a subset of HCC-free patients [13, 15]. Nonetheless, AFP elevations are associated with greater long term risk of HCC development [16].

The practical use of AFP is complex and suboptimal due to varying recommendations on the upper limit of normal (ULN) with associated variability in sensitivity and specificity depending on the ULN applied [11] which may vary between aetiologies of the underlying liver disease, ethnicity and sex [13, 17–19]. The absence of better serum biomarkers and the problems of cumulative radiation exposure and cost associated with cross sectional imaging leaves ultrasound (US) as the currently recommended sole modality of surveillance [9, 10]. Unfortunately, as a screening test, US itself is suboptimal in its sensitivity and specificity for HCC detection [13, 20]. Although AFP has been removed from updated international guidelines for HCC surveillance, a number of recent reports propose a rationale for the ongoing use of AFP [21–23].

Refining AFP use through alternative methodologies for AFP interpretation has been proposed to improve HCC detection and possibly cost efficiency [15, 17, 23–27]. These studies rely on operator interpretation of AFPs, often over a specified time scale, which is likely to create difficulties in the real world where there is already a wide variation in how HCC surveillance is practised even by specialist clinicians [28]. Furthermore, gaps in knowledge about HCC surveillance in practice have been identified as a frequent factor leading to failure to engage primary care physicians in HCC surveillance programmes [29–31]. Therefore, a potential improvement could be the development of an automated personalised interpretation of an individual patient’s AFP screening results capable of operating adaptively over varying screening intervals.

Despite the removal of AFP from surveillance guidelines, monitoring of the HCC-risk population in Lothian, South East Scotland, continued to employ local guidelines of bi-annual surveillance using US and AFP, aided by the graphical display of AFP levels over time in a patient’s record from a dedicated tumour marker database. For both all newly diagnosed HCC cases and a large cohort of patients in HCC-surveillance in 2009 (for whom all AFP results were available) we investigated the role of AFP in the clinical pathway leading to HCC diagnosis and whether formal automated analysis of dynamic AFP can facilitate HCC detection and enable curative treatment. Here, in a proof of principle study, we show that specialist interpretation of dynamic AFP changes over time can be modelled and a standardised automatic algorithm devised to identify early a subset of patients at higher risk of HCC development.

## Methods

### Patient identification and data retrieval

Lothian is a distinct region of South East Scotland providing universal healthcare for approximately 800,000 inhabitants. Coordinated care is facilitated by unified secondary healthcare records and a unique individual patient identifier: Community Health Index (CHI). Anonymised patient data were collected and analysed under local ethics approval granted by South East Scotland Research Ethics service (Caldicott ref NR/1201AB15). A dedicated regional hub (Royal Infirmary of Edinburgh) receives all regional referrals of suspected HCC for diagnosis, and their management is guided in accordance with local guidelines at a regional multidisciplinary meeting. On-site regional interventional radiology, tertiary hepatobiliary surgical and the national liver transplant services are available.

### Serum AFP detection

Serum samples throughout the region are collected in a unified blood collection system and analysed in a central laboratory using ADVIA Centaur XP Immunoassay System (Siemens) or Architect AFP assay (Abbott Laboratories) which were validated for comparability. Results are reported as kU/L (Conversion factor 0.83kU/L = 1ug/L).

### Review of HCC cases

Records for all referrals of suspected HCC from October 2008 to February 2015 were reviewed by a local specialist (TGB) to identify newly diagnosed HCC cases, cross-referenced with ICD-10 codes and a local liver transplant database. Diagnosis of HCC was based upon international guidelines for imaging and histopathological criteria [8, 9]. Referred to hereafter as the ‘*HCC case series’*, data were retrieved as follows: survival status at census date, patient age at diagnosis, aetiology of chronic liver disease, sex, AFP dates and results, imaging modality dates and results, pathological sample reports and dates. All interventional therapies and procedures (liver transplant, surgical resection by partial hepatectomy, trans-arterial chemoembolization, radiofrequency ablation, and/or Sorafenib therapy) and associated dates were recorded together with size (mm) and number of HCC lesions at the time of diagnosis. Detailed review of electronic case-notes established the clinical path by which HCC was detected. The date of HCC diagnosis was defined as the date of an imaging procedure, biopsy or surgery which led to the first diagnosis of HCC in each patient. Patients with HCC diagnosed between 1^st^ January 2009 and 31^st^ December 2014 were included in the analyses. Cases were excluded whose original referral was outwith the Lothian region, or if the radiological diagnosis of HCC was in doubt or excluded subsequently by histology. Data detailing AFP values in this cohort have been deposited in Edinburgh DataShare: http://dx.doi.org/10.7488/ds/1395.

### HCC surveillance cohort: identification

A separate cohort of patients undergoing intentional HCC surveillance with AFP was identified. Local guidelines throughout the study period recommended ongoing use of 6 monthly AFP and abdominal US. A local tumour marker database consisting of all AFP samples collected within the region was interrogated for patients receiving ≥2 AFP levels within a 24 month period (samples ≥3 months apart) with at least one AFP measurement in 2009. Exclusion criteria were age <18 years, suspicion of germ cell tumour, no recorded indication for HCC surveillance as outlined by international guidelines [9, 10], and previous HCC. After rigorous data-checks on an initial database, 1,509 patients with 14,842 AFP readings met inclusion criteria, forming the ‘HCC surveillance cohort’ with data through March 2012 in all instances. For individual cases, the data retrieved and anonymized were: age in 2009, sex, aetiology of liver disease, AFP dates and results. Aetiology was coded as: alcoholic liver disease (ALD), Hepatitis C virus (HCV), non-alcoholic fatty liver disease (NAFLD), Hepatitis B virus (HBV), primary biliary cholangitis (PBC), haemochromatosis (Haem), Autoimmune liver disease (AILD), and other. Additional prospective data on specific trigger-case (see later) and control sets were retrieved as described subsequently. For calculation of HCC-free survival, follow-up time was calculated from first AFP measurement date in 2009 to either i) last census date prior to April 2012 (which comprised the dataset for full analysis) or ii) date of diagnosis of HCC. Overall, HCC development rate was 1.67 HCC per 100 patient years (75,933 follow up days for 59 cases where HCC developed and 1,215,639.5 days for 1,509 HCC-free cases allowing for 131.5 days extra per person [half the mean interval between AFPs of 263 days—see results]). Follow up days were determined by time from first AFP reading after 01/01/2009 and either HCC diagnosis or last AFP prior to census of AFP database (31/03/2012). Data have been deposited in Edinburgh DataShare: http://dx.doi.org/10.7488/ds/1397.

### Exploratory analysis of the finalized AFP-database

Analyses were stratified by aetiology, and particular interest focussed on a sub-cohort of 1048 patients with at least six AFPs. We assessed linearity of log_10_-AFP (hereafter log-AFP) against historical-time, back from the most recent screen, so that patient-intercepts were our best estimate for ‘true’ log-AFP at the patient’s most recent AFP-screen. Linearity was tested for all available screenings; and for the most recent six.

Per-patient, whether a linear trend adequately described the patient’s time-ordered log-AFPs was summarised by R^2^ (the proportion of variation in log-AFPs explained by linearity). Within each aetiological group, we focused on patients for whom linearity explained at least 30% of the variation in their log-AFPs. However, it also mattered if the estimated gradient was negative, indicating increasing AFP-levels to the most recent.

### Bayesian analysis plan

Initial insights: The Bayesian analysis first contrasted linearity for the four major diagnostic groups by analysing only those patients for whom there was adequate support for linearity either on the basis of all, or the six most recent, log-AFPs. This initial analysis, which fitted an indicator for early-diagnosed HCC cases, confirmed that the log-AFP intercept was highest and gradient most strongly negative for early-diagnosed HCC cases; with the HCV sub-cohort next in line.

The initial Bayesian set-up allowed estimation of parameters (such as aetiological-mean intercepts) when based on all screened log-AFPs or when windowed-in on the most recent six. Aetiology-specific standard deviations measured how variable patient-specific estimates were about their aetiological-means—whether in terms of intercept or gradient.

### Definitive Bayesian analysis: entry criteria

Individuals were included in this analysis if they met the following criteria: (i) at diagnosis the HCC was considered 'potentially curable', as defined by current UK liver transplantation criteria [32, 33]; (ii) at least six AFPs were available; (iii) the liver disease aetiology was one of the four main aetiology groups—ALD, HCV, NAFLD, and HBV; and (iv) at any point in their chronology, a patient’s most recent six log-AFPs, or all log-AFPs up to and including the most recent, provided an R^2^ greater than 0.3.

### Bayesian analysis: static and dynamic modes

Ultimately, we needed to apply our Bayesian analysis dynamically once a patient had achieved six or more AFPs and sufficient evidence of linearity, as per the entry criteria. Early-diagnosed HCC cases sit within their aetiology, rather than have a separate indicator. The training data-set, albeit dynamically-defined, was initially analysed in respect of the maximal AFP trajectories for those who had met the entry criteria (that is: in static mode) to determine trigger-regions, defined by patients’ estimated log-AFP intercept and gradient, in which most of the early-diagnosed HCC cases were located.

The training data-set was then analysed dynamically (from wave 1 to the final wave W, which coincides with the static analysis, see S1 Supporting Methods) to establish the likely recall/re-screening burden occasioned by possibly-repeated triggering as a patient’s data accumulated. Additional methodological description of the Bayesian analysis is provided in S1 Supporting Methods. The Bayesian hierarchical linear model, which underlies our dynamic Bayesian algorithm, was fitted using OpenBUGS software [34].

### Case controls analysis

Case-controls analysis was performed using 32 cases which triggered our dynamic Bayesian analysis. For each trigger-case and trigger-year, five controls matched for i) aetiology, ii) sex, iii) age to within five years and iv) ongoing AFP screening to within 1 calendar-year of trigger-case year, were randomly selected by PD and SMB; and outcome information during the 2009–2014 was gathered by TGB. As trigger-cases could signal in more than one calendar-year, we present information from controls for the earliest trigger-year only.

By design, the ‘*HCC surveillance cohort’* excluded patients who were HCC-diagnosed prior to 2009. The calendar-year of first-triggering by the listed trigger-cases may have been pre-2009 but the case-control assessment is based on person-months at-risk from the later of January 2009 or first trigger-year. The count of person-months is terminated by the earliest of three events: HCC diagnosis, OLT (orthotropic liver transplant) or death, with the month of the event’s occurrence counted as an at-risk month.

## Results

### Part 1

#### Epidemiology of HCC development in a loco-regional Scottish cohort

**Fig 1:**
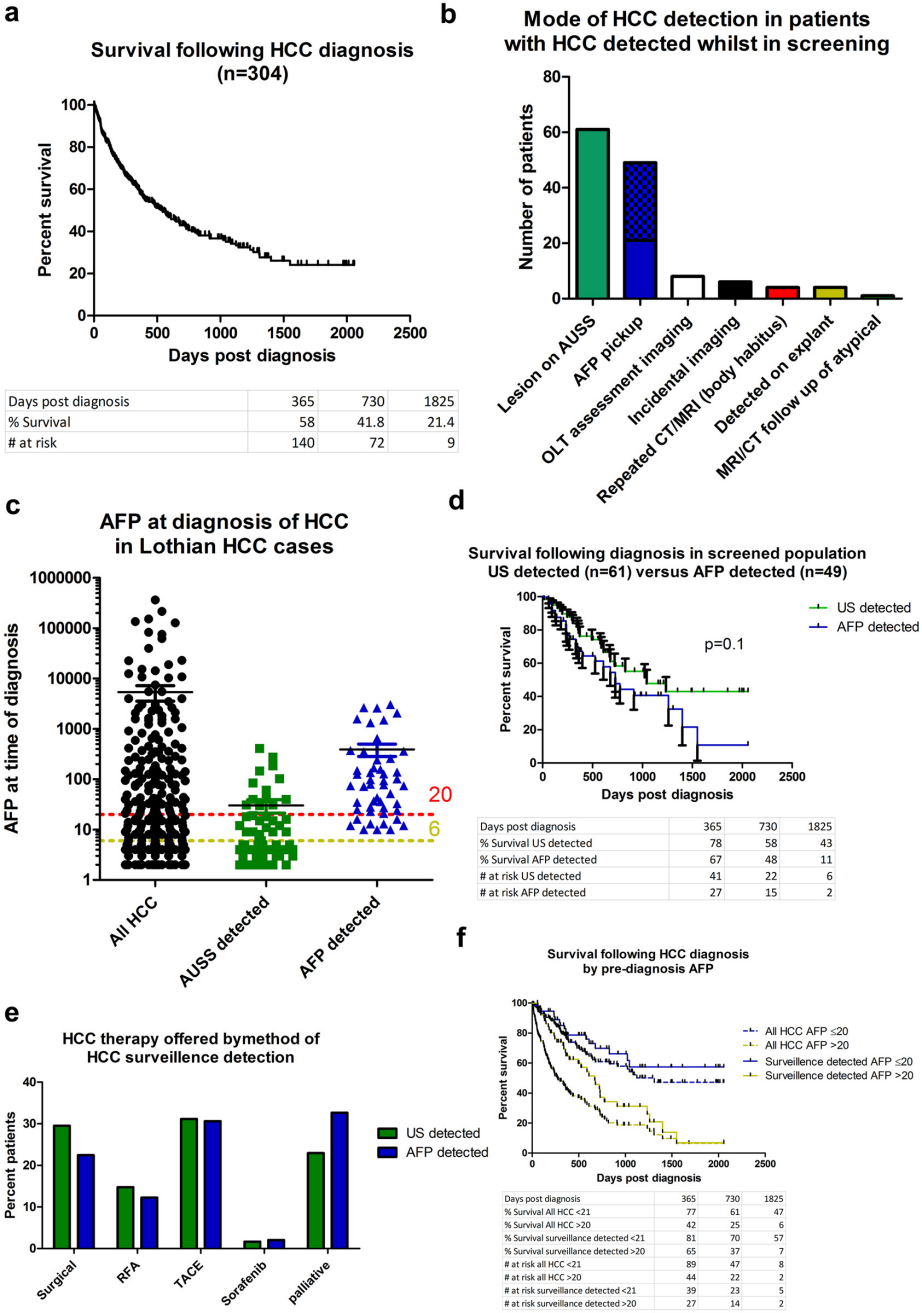
AFP as an HCC surveillance tool detects a significant number of treatable HCC in patients with satisfactory outcomes. **A**) Survival for total HCC cohort diagnosed with HCC between 1/1/2009 and 31/12/2014. **B**) The role of AFP in HCC detection. Method of HCC detection for the 133 patients within HCC surveillance programme at the time of diagnosis, chequered area within AFP pickup group represents the 28/49 patients in whom a recent US had not been performed—see text for details. **C**) Individual AFP levels at time of diagnosis for patients diagnosed with HCC, AFP values plotted at log10; AFP = 6 (local ULN; yellow) and AFP = 20 (red) are shown. All columns p<0.0001 to one another by Kruskal Wallis test with Dunns multiple comparison. **D**) Survival of patients with HCC diagnosed through surveillance screening either through US or AFP mediated conversion to CT/MRI imaging, error bars = SEM, p value denotes Mantel Cox. **E**) Therapy offered to patients within each group (US detected n = 61 and AFP detected n = 49) of patients with HCC detected during surveillance; all p>0.05 by 2 way Anova. Of the 11 and 9 patients listed for liver transplantation, 2 (due to tumour growth) and 1 (due to frailty) were delisted from the waiting list whilst awaiting transplantation in US and AFP detected groups respectively.

During the six years of 2009–2014, 304 new cases of HCC were diagnosed in the Lothian region, 77% of whom were male. Median age at diagnosis was in the seventh decade, see Table 1. The most frequent aetiologies of cirrhosis were ALD, NAFLD and HCV. Median survival following HCC diagnosis was 539 days; survival at 1, 2 and 5 years post-diagnosis was 58%, 42% and 21% (Fig 1a). Tumour characteristics at diagnosis are shown in S1 Table.

**Table 1:** Demographics and route to diagnosis in HCC patients.

**A**		
**Variable**	HCC n = 304	
**Sex/male (%)**	235 (77)	
**Age**†	68 (17–98)	
Male	68 (17–98)	
Female	69 (33–88)	
Aetiology/% (*)		
Alcohol related liver disease	21.7 (37.5%)	
Non alcohol related fatty liver disease	19.7 (26.3%)	
Hepatitis C	13.8 (21.7%)	
Hereditary haemochromatosis	2.6 (4.6%)	
Hepatitis B	3.3 (4.3%)	
**B**		
**Total HCC**	n = 304	100%
**Index event triggering diagnosis**		
Screening	**133**	43.80%
Standard optimal (‡)	90	29.6% (1.6%)
Suboptimal	25	8.20%
Detected incidentally out with screening (††)	14 (8)	4.6% (2.6%)
Detected only on transplant explants	4	1.30%
Symptomatic consistent with HCC	67	22.00%
Presentation (‡‡)	53 (4)	17.40%
Incidental finding on imaging	47	15.50%

Aetiology is given for the 5 most prevalent. Percentages apply for cases where only a single aetiology was defined; bracketed percentages refer to the total of cases where aetiological factor was either alone or as a cofactor. Other aetiologies were %; AIH 2.3 (2.3), PBC 3.0 (3.3), PSC 0 (0), Hepatic sarcoid 0.3 (0.3), Secondary haemochromatosis 0.3 (0.3), Confirmed non cirrhotic 1 (1), Cryptogenic cirrhosis 15.5. (% total of sole aetiology and as cofactor).

^†^Median (range),

*Sole aetiology (total of sole and cofactor),

^‡^Optimal, but CT/MRI due to habitus,

^††^incidental at OLT assessment,

^‡‡^Representation to liver clinic.

#### Clinical events leading to the detection of HCC

Detailed case review established the clinical events leading to HCC diagnosis. The largest single reason for HCC detection was participation in an HCC surveillance programme which accounted for 44% (n = 133) of all detected HCCs (Table 1). However, 14 (11%) of these had their HCC detected by investigations outwith routine screening: detection in eight patients was during assessment for liver transplantation for decompensated liver disease, two had hepatological indications for repeat imaging (decompensation and portal vein thrombosis) and four patients received either CT or MRI for other indications (altered bowel habit; assessment of Crohn’s disease; weight loss following acute myocardial infarction; weight loss and altered bowel habit with history of colorectal carcinoma). Despite screening, a further four patients had no known HCC until liver explant examination confirmed tumour; of these, three had maximal tumour size of 1cm (1, 2 and >5 lesions) with the other patient having extensive multifocal disease (maximum diameter 63mm). Of the remaining 115 surveillance-detected HCCs, the majority (n = 90) had 6-monthly imaging and AFP; referred to as optimal screening. Five of this optimally screened group had regular cross sectional imaging (CT/MRI with contrast) because of insufficient US views due to body habitus. The other 25 patients had less regular HCC screening investigations, by reason of failure to attend or unexplained omission of screening by clinicians; termed suboptimal screening.

Symptoms consistent with HCC development (weight loss, right upper quadrant pain, itch, hepatic decompensation, or pain/effusion consistent with sites of metastasis) accounted for 22% of all detected HCCs (n = 67). Fifty-three HCCs (17%) were detected by investigation of patients referred for abnormal LFTs, hepatomegaly, newly diagnosed HCV or for investigation of symptoms not consistent with underlying HCC. A further four patients (1.3%) had HCC detected at re-engagement with specialist services having been previously lost to follow up. Finally, 47 HCCs (16%) were detected incidentally on imaging performed for other reasons including aortic aneurysm surveillance, assessment of peripheral vascular disease, haematuria, dyspnoea, and entry into research studies.

#### The role of AFP surveillance HCC detection in our patients

From clinical records we ascertained the contribution of AFP to HCC diagnosis for 129 patients undergoing HCC surveillance (excluding the four incidental tumours found at liver transplant explant analysis).

Ultrasound (US) detected an initial lesion in 61 cases (48%), of whom 59 had an AFP level taken within 6 months of diagnosis (Fig 1b). Twenty-nine (48%) had raised AFP levels (local upper limit of normal [ULN] ≥6kU/L) at diagnosis (Fig 1c).

Detection of HCC was frequently because clinical interpretation of AFP led to altered management resulting in HCC diagnosis. This occurred in 49 of the 129 (38%) patients whose HCC was detected whilst in an active screening programme (Fig 1b) and whose sole indication for conversion to cross-sectional imaging had been the rising AFP. Twenty-one of the 49 had an US within 6 months prior to HCC-diagnosis which had failed to identify any suspicious lesion. Eighteen of the 49 cases (37%) were in optimal HCC surveillance with long-term regular 6-monthly US and AFP. Details of the other 28 cases with AFP-flagged HCC diagnosis in whom no recent US imaging was performed are given in S2 Table. Median AFP at HCC-diagnosis in this group of 49 patients was 77kU/L (Fig 1c and S1 Table).

#### Outcomes for patients by the modality of HCC detection

Due to the importance of surveillance to detect HCC with a potentially favourable clinical outcome, we analysed the survival of HCC patients detected by AFP versus US. Median survival for 49 AFP-detected HCC was 729 days post-diagnosis compared to 1043 days in 61 patients with US-detected HCC, but the difference in survival was not statistically significant (Fig 1d).

The aim of surveillance is to facilitate intervention for early stage HCC. At diagnosis, 78% and 65% in the US and AFP detected groups respectively met UK liver transplantation criteria for size and number of HCC lesions (S1 Table). Applying the additional, current UK liver transplantation criterion, for an upper AFP limit of 1000kU/L, zero and four patients in the US and AFP detected groups respectively became ineligible. All four cases excluded by an AFP >1000kU/L had solitary tumours of size 25, 27, 30 and 45mm.

To assess if detection of HCC by AFP was futile with regard to treatment options, specific treatments actually received for HCC were compared: 44% (27/61, 95% CI: 32%-57%) of patients with HCC detected by US were offered a potentially curative therapy (either resection, RFA or liver transplantation) compared to 35% (17/49, 95% CI:22%-48%) of patients with AFP detection (Fig 1e and S1 Table); 23% and 33% respectively were offered best supportive care; 29% and 35% received transarterial chemo-embolization with doxorubicin/lipiodol (TACE) monotherapy, often in repeated sessions. In summary, 75% and 65% of the US and AFP detected HCCs received surgical/interventional radiological therapy, confirming that detection of HCC through the use of AFP does not result in futile detection of untreatable HCC.

#### Patient outcomes by AFP at HCC diagnosis

Elevated AFP has been associated with poorer outcome following HCC diagnosis, particularly around the level of >20kU/L, often used as a decision limit [35–37]. We therefore examined the effect of AFP at diagnosis on patient survival. In the whole HCC cohort, a cut-off of AFP>20kU/L separated cases with differing survival (Fig 1f). This separation was preserved when applied to the cases detected by either US or AFP within a screening cohort. Nine of the 49 cases (18%) detected solely by AFP fell below this cut-off. Therefore, in this study, an individual patient’s outcome was dependent on the AFP value at diagnosis and, if AFP is to be used in HCC surveillance, early recognition of a rising AFP may be crucial to prevent the patient entering a poorer prognostic group.

#### Serial AFP look-back in the ‘HCC case series’

Detailed review of the clinical records of patients whose HCC detection was prompted by a rising AFP revealed that, in most cases, elevation above a baseline for that patient prompted a conversion (often temporary) to CT/MRI based imaging over US. Locally, clinicians were provided with a graphical representation of AFP values over time with each AFP result. We hypothesised that such use of graphically presented serial AFPs aided clinicians’ interpretation of the relevance of an individual’s recent versus past AFPs and that a combination of current value and rate of increase were key features in their decision making process.

**Fig 2:**
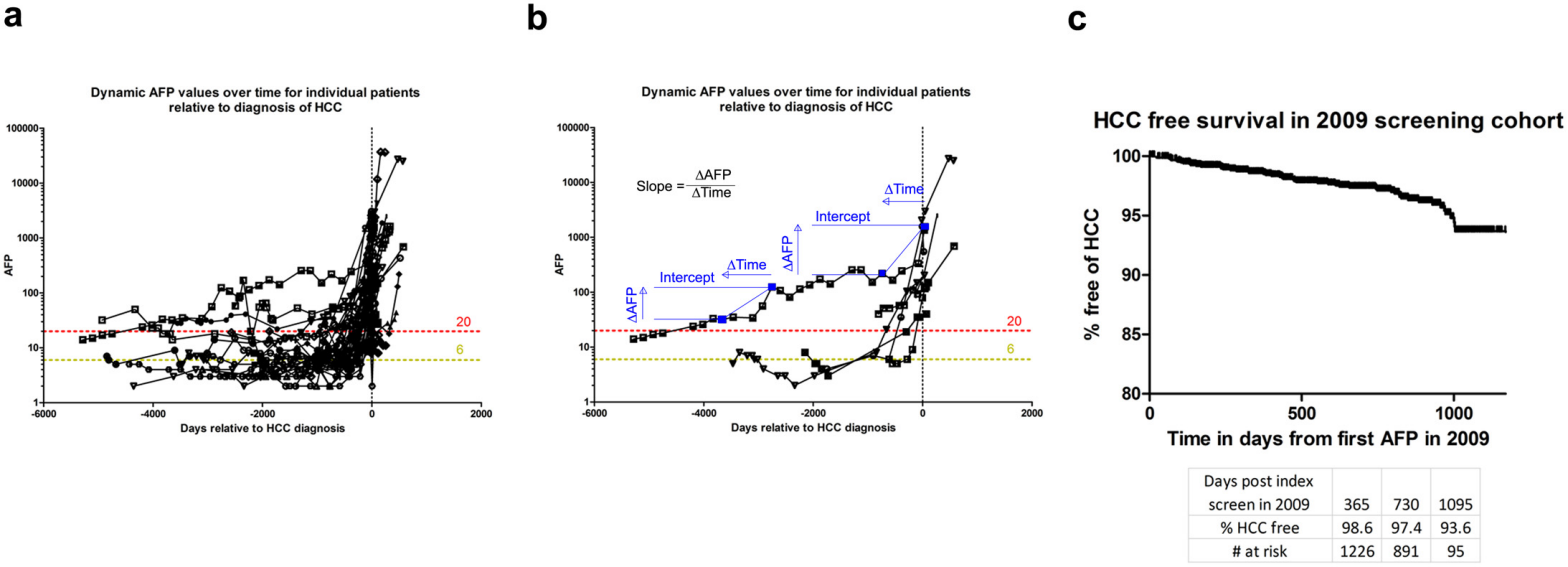
Dynamic AFP changes associated with HCC development. **A**) Plotting each individual’s time course of serum AFP relative to diagnosis an elevation in values is observed prior to diagnosis. Here all individuals in the HCC review in whom AFP influenced clinical management (n = 49) are charted with AFP on a log_10_ scale. **B**) Graphic demonstrates the concept of gradient and intercept over a specific individual’s time course. **C**) The screening cohort of 1509 patients followed over time for development of HCC, overall incidence at end of index screening (1186 days from 01/01/2009, total HCC free % = 93.7).

Examining all patients with ‘AFP detected’ HCC reveals an apparent inflection of AFPs prior to their diagnosis, consistent with our hypothesis (Fig 2a). Examining individual cases more closely, this effect becomes more apparent (Fig 2b). None the less the decisions to alter patient management based upon AFP variations were not standardised, sometimes inconsistent and difficult to put into agreed protocol due to the complexity of recognising what was considered ‘at risk’ versus ‘not at risk’. We therefore set out to assess more rigorously the case for monitoring how serial AFPs evolve and describe the analysis of serial AFPs in untutored observer fashion, in order to establish a standard for interpretation of dynamic AFP results.

### Part 2

#### The ‘HCC surveillance cohort’

To investigate further the role of AFP in HCC detection within our region, we identified patients undergoing HCC surveillance in 2009; termed ‘HCC surveillance cohort’. The year, 2009, was chosen so that all patients developing HCC could be cross-referenced to our HCC cases. In total, 1509 patients were identified by predetermined criteria—see **Patients and Methods** section. Eighty seven of these patients developed HCC by January 2015, 59 by the analysis-date of 31 March 2012 which corresponds to an average HCC development rate of 1.67 per 100 patient years (Fig 2c).

#### Exploratory analysis of the ‘HCC surveillance cohort’ and ‘HCC case series’

**Fig 3:**
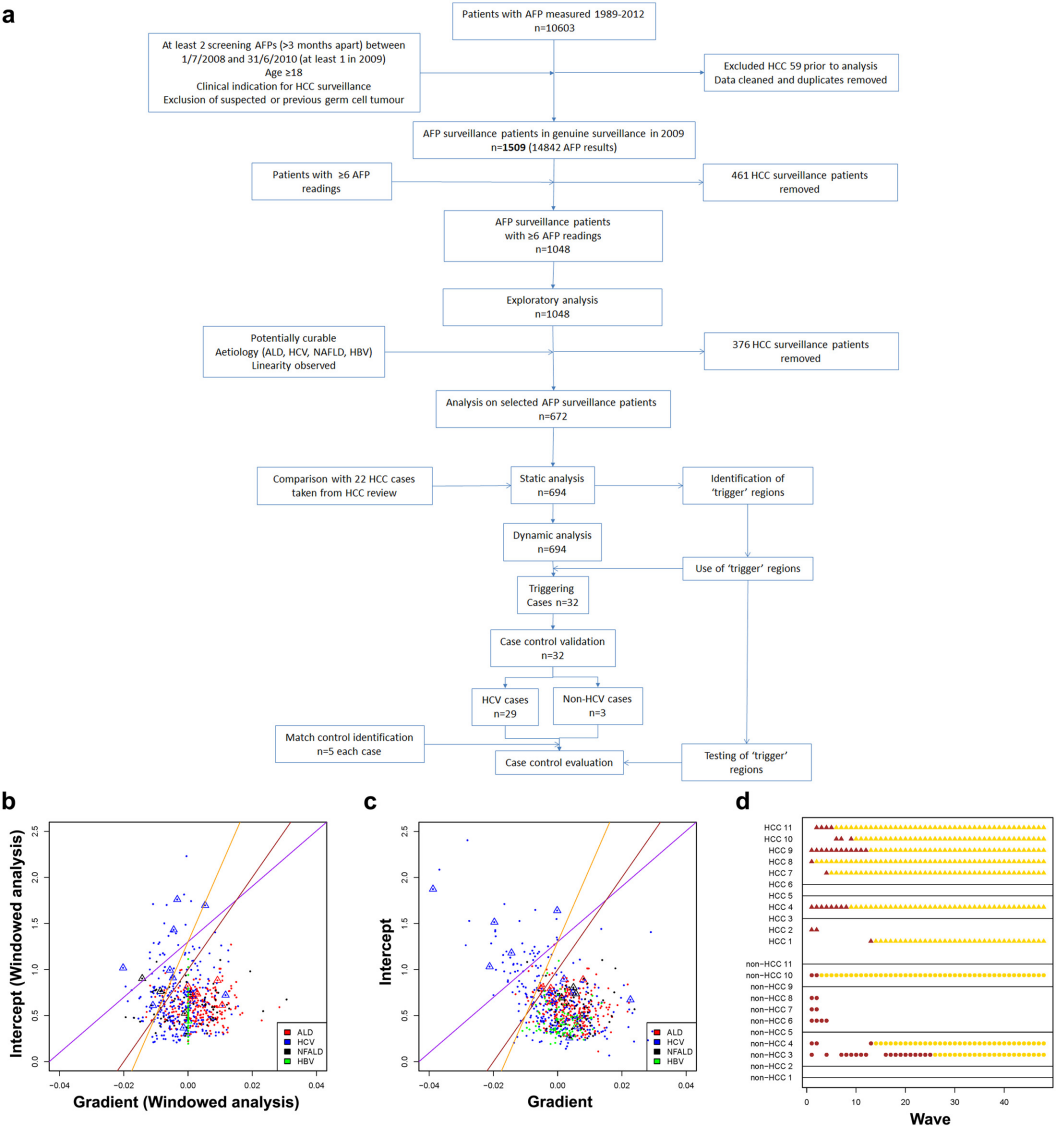
Using dynamic analysis of AFP provides a methodology for identifying patients at high risk of HCC. **A**) Workflow for the development of an algorithm for HCC detection using AFP. The *HCC surveillance cohort* refined to patients with specific characteristics prior to formal Bayesian analysis in static and dynamic modes. In static mode a trigger zone was established, which was then tested dynamically. Estimated patient-specific intercept and gradient parameters plotted against each other. Estimates were taken from `windowed' version **B**) `full-data' version **C**) of full-trajectory retrospective Bayesian analysis. Triangles denote confirmed early-diagnosed HCC cases. Diagonal lines define regions of parameter space (above the line) that might indicate emerging HCC cases: purple—passes through (x, y) = (-0.01, 1) and (0, log20); brown—passes through (x, y) = (-0.01, 0.5) and (0, 1); yellow—passes through (x, y) = (-0.01, 0.5) and (0, log20). *The area to the above/left of the yellow line was used to represent the area of ‘high risk’ characteristics of AFP*. **D**) Illustration of triggering across waves of prospective Bayesian analysis. All HCC patients from the HCV group are shown, along with an equal number of non-HCC cases from the same group. A point is plotted for each trigger (HCCs denoted by triangles and non-HCCs by circles); a horizontal line is shown for patients who did not trigger at all. Points of a lighter shade are used to indicate that the patient-specific data are the same as in the preceding wave due to that patient's data set having ceased to accrue more AFPs in the training data-set.

Having two separate data sets (one comprising all HCCs diagnosed within the region, the other a prospective cohort of HCC-free patients in HCC screening) combined with all AFP results and dates in both sets, the work flow Fig 3a shows how, after rigorous data-checks, we utilized the available serial AFP values for 1509 validated patients in the HCC surveillance cohort to devise a standardised algorithm for detecting high risk AFP values for an individual. We elected to use a Bayesian approach which allows interpretation of an individual’s AFP history to draw strength from the history of others from the same aetiology. This method attaches a posterior probability to the intercept and the gradient of a log-AFP history lying within a trigger region to identify high HCC risk.

The two most common aetiologies in Lothian’s *HCC surveillance cohort* of 1509 patients were ALD (426 patients) and HCV (406 patients). The cohort was predominantly male (57%) but, for two aetiologies in particular, PBC (101 patients) and AILD (80 patients), females predominated: 88% and 80% respectively (Table 2). Mean age in 2009 was 55 years but was higher in ALD patients (58.8 years, se 0.5 years) than for HCV patients (48.2 years, se 0.5 years).

**Table 2:** Exploratory analysis of HCC surveillance cohort.

	Total *'HCC surveillence cohort'*						'*HCC Surveillence cohort'* with ≥6 AFP samples						'*HCC surveillance cohort'* for analysis					
Aetiology	Patients	Males, %	Ln AFP: mean (sd).		Age in 2009	Screens per-case	Patients	Males, %	Ln AFP: mean (sd).		Age in 2009	Screens per-case	Patients	Males, %	Ln AFP: mean (sd).		Age in 2009	Screens per-case
			Last screen	First screen	Mean (sd); n	Mean (sd).			Last screen	First screen	Mean (sd); n	Mean (sd).			Last screen	First screen	Mean (sd); n	Mean (sd).
**All patients below**	**1509**	860, 57	1.29 (0.63).	1.36 (0.71).	55.1 (12.9); 1494	9.8 (6.8).	**1048**	599, 57	1.33 (0.65).	1.38 (0.72).	56.3 (11.9); 1040	12.6 (6.4).	**672**	440, 66	1.38 (0.69).	1.45 (0.72).	55.5 (11.0); 668	13.6 (6.8).
**ALD**	426	283, 66	1.35 (0.54).	1.46 (0.56).	58.8 (9.8); 421	9.3 (5.8).	299	194, 65	1.35 (0.56).	1.46 (0.56).	59.4 (9.5); 298	11.7 (5.3).	252	168, 67	1.33 (0.54).	1.47 (0.55).	59.9 (9.7); 251	12.5 (5.3).
**HCV**	406	280, 69	1.42 (0.79).	1.38 (0.83).	48.2 (10.3); 403	11.8 (8.6).	304	210, 69	1.51 (0.82).	1.43 (0.85).	49.9 (9.4); 302	14.5 (8.2).	267	182, 68	1.56 (0.84).	1.48 (0.87).	50.1 (9.1); 265	15.4 (8.4).
**NAFLD**	159	93, 59	1.25 (0.53).	1.42 (0.57).	64 (10.3); 159	7.4 (4.6).	101	60, 59	1.23 (0.48).	1.4 (0.55).	65.7 (9.2); 101	9.6 (4.3).	68	41, 60	1.3 (0.49).	1.55 (0.54).	65.7 (9.3); 68	10.7 (4.8).
**HBV**	157	78, 49	1.08 (0.55).	1.17 (0.68).	46.5 (11.7); 154	9.7 (5.7).	110	58, 53	1.05 (0.52).	1.16 (0.69).	49.5 (10.7); 108	12.3 (5).	85	49, 58	1.06 (0.55).	1.19 (0.75).	51.1 (10.3); 84	13.4 (5.0).
**PBC**	101	12, 12	1.31 (0.58).	1.36 (0.59).	63.8 (10.0); 100	10.3 (5.9).	77	8, 10	1.31 (0.58).	1.36 (0.59).	64.4 (9.7); 76	12.3 (5.2).						
**Haem**	86	63, 73	1.07 (0.52).	1.04 (0.63).	53.2 (12.3); 85	7.8 (4.3).	54	38, 70	1.17 (0.51).	1.13 (0.62).	57.4 (11.6); 53	10.1 (3.9).						
**Auto-immune**	80	16, 20	1.18 (0.47).	1.46 (0.98).	59.3 (15.2); 78	12.9 (7.5).	62	14, 23	1.19 (0.48).	1.51 (1.0).	60.7 (13.9); 61	15.5 (6.4).						
**Other**	94	35, 37	1.23 (0.59).	1.2 (0.73).	55.3 (18.2);94	6.7 (5.3).	41	17, 42	1.2 (0.52).	1.22 (0.77).	53.9 (18.1); 41	11.3 (5.2).						

Three separate cohorts were analysed, left—the total 1509 patients within the ‘*HCC surveillance cohort*’, centre—the 1048 patients of the 1509 with ≥6 AFP values, and right—the 672 patients of the 1048 from the 4 major aetiologies (HCV, ALD, NAFLD or HBV) who showed evidence of linearity (R^2^>0.3) for inclusion in Bayesian analysis.

Mean of log-AFP at the patient’s most recent screen was similar at 1.35 (se 0.026; back transformed 5^th^ and 95^th^ centiles for AFP 2.9 to 173) for ALD patients and 1.42 (se 0.039; back transformed 5^th^ and 95^th^ centiles for AFP 1.3 to 524) for HCV patients. However the standard deviation (sd) for log-AFP at both first and most recent screen was much higher for HCV patients (around 0.80) than for ALD patients (around 0.55); and, for ALD patients but not for HCV patients, mean log-AFP had decreased from first to most recent screen.

Table 2 also presents equivalent data values for the 1048 patients which at least six AFPs and the 672 patients in the four main aetiologies whose AFP trajectory gave sufficient evidence of linearity to admit them to the definitive Bayesian analysis (see below).

Prior to definitive Bayesian analysis, three checks were necessary, these and their results are summarised in S3 Table. With the data having passed all three of our preliminary checks we proceeded to the definitive Bayesian analysis.

#### Bayesian analysis (in waves) of the ‘HCC surveillance cohort’ and ‘HCC case series’

We let p_i_ denote the "point of entry" (into our dynamic analysis) for individual i. Traversing his/her data chronologically, “point of entry” is defined as the observation number at which his/her log-AFPs first provided an R^2^ greater than 0.3 for linearity, either over the most recent six AFPs or for all AFPs up to and including the most recent.

Our dynamic analysis comprised 48 waves, indexed by w. For wave 1, our data-set comprises all AFPs up to (and including) the point of entry for each individual. We then consider that all individuals are `synchronised' at this point with subsequent AFP measurements also arriving in synchronised fashion. The data-set grows in each subsequent wave such that a single additional AFP reading is included for each individual, if available (Fig 3d).

In static mode, our Bayesian algorithm analyses only the final (wave 48) data-set. But, dynamically, the analysis runs at each wave of data-acquisition (1 to 48). For each aetiology (denoted by k), the algorithm allows intercepts and gradients to differ randomly across patients.

$$\begin{array}{c} y_{ij}=\sum_{k=1}^{4}\alpha_{{}_{i}}^{k}x_{ik}+\sum_{k=1}^{4}\beta_{{}_{i_{}}}^{k}x_{ik}\left(z_{ij}/100\right)+e_{ij}\\ \alpha_{i}^{k}\sim N\left(\mu_{\alpha }^{}{}_{k},\sigma_{\alpha }^{2}{}_{k}\right),\beta_{i}^{k}\sim N\left(\mu_{\beta k},\sigma_{\beta k}^{2}\right),e_{ij}\sim N\left(0,\sigma_{i}^{2}\right) \end{array}$$

In the equation above, *y*_*ij*_ denotes log-AFP for patient i at measurement time j, the covariate *z*_*ij*_ represents number of days since last screening and the indicator *x*_*ik*_ takes the value 1 if patient *i* has aetiology *k*, zero otherwise. The model is explained in more detail in the S1 Supporting Methods section.

In static mode, the algorithm allowed us to specify trigger regions which appeared to identify patients at high risk of HCC development. We tested the chosen trigger region (coloured yellow, Fig 3b and 3c) and 0.67 posterior probability threshold by analysing the HCC surveillance cohort data dynamically. This identified 32 cases who triggered (often repeatedly) high risk AFP behaviour. Individual graphs for these cases are presented in S1 Fig.

Table 3 shows the algorithm’s parameter estimation at wave 1 and wave 48, each analysis pertaining to 672 patients from HCC surveillance cohort and their 22 early-diagnosed HCC counterparts. Median intercept is highest (0.68 at wave 48) and the gradient most negative for the HCV patients. Comparison between waves 1 and 48 shows, for the windowed analysis especially, the extent of learning about median gradient which for HCV patients was from -0.001 at wave 1 to -0.003 by wave 48.

**Table 3:** Model coefficients obtained from full-data and windowed analyses in static mode (wave 48) and dynamic mode (wave 1).

Parameters in the Bayesian algorithm	Intercept for the aetiological population (most recent log10-AFP)			Intercept for the individual (sd. of individuals about their aetiological mean)			Gradient for the aetiological population			Gradient for the individual (sd. of individuals about their aetiological mean)		
	*μ*_*α*_			σ_*α*_			*μ*_*β*_			σ_*β*_		
	Median	2.50%	97.50%	Median	2.50%	97.50%	Median	2.50%	97.50%	Median	2.50%	97.50%
**Full-data analysis, Wave48**												
ALD	0.58	0.55	0.6	0.19	0.17	0.21	0.004	0.003	0.005	0.008	0.007	0.009
HCV	0.68	0.64	0.72	0.37	0.34	0.40	-0.001	-0.003	0.0003	0.011	0.010	0.013
NFALD	0.57	0.52	0.63	0.21	0.18	0.26	0.006	0.003	0.008	0.008	0.005	0.011
HBV	0.45	0.41	0.49	0.17	0.15	0.21	0.001	-0.001	0.002	0.006	0.004	0.007
**Windowed analysis, Wave48**												
ALD	0.57	0.54	0.59	0.18	0.17	0.20	0.003	0.001	0.005	0.010	0.007	0.012
HCV	0.68	0.64	0.72	0.35	0.31	0.38	-0.003	-0.005	-0.001	0.010	0.008	0.013
NFALD	0.59	0.54	0.64	0.2	0.17	0.25	0.0002	-0.004	0.004	0.012	0.008	0.018
HBV†	0.44	0.4	0.48	0.18	0.16	0.21	0.0001	-0.001	0.001	-	-	-
**Full-data analysis, Wave1**												
ALD	0.61	0.58	0.64	0.18	0.16	0.20	0.001	0.0001	0.003	0.005	0.003	0.006
HCV	0.66	0.62	0.7	0.28	0.25	0.32	-0.001	-0.002	0.001	0.008	0.007	0.009
NFALD	0.56	0.49	0.62	0.23	0.19	0.29	0.007	0.004	0.011	0.010	0.007	0.014
HBV	0.48	0.43	0.54	0.2	0.16	0.24	-0.0003	-0.002	0.002	0.006	0.004	0.008
**Windowed analysis, Wave1**												
ALD	0.60	0.57	0.63	0.19	0.17	0.22	0.003	0.001	0.004	0.010	0.008	0.012
HCV	0.67	0.63	0.72	0.30	0.27	0.35	-0.001	-0.003	0.001	0.011	0.009	0.013
NFALD	0.54	0.47	0.61	0.25	0.20	0.31	0.010	0.005	0.014	0.015	0.012	0.020
HBV†	0.44	0.38	0.51	0.20	0.17	0.25	0.001	-0.002	0.004	-	-	-

Posterior medians and 2.5%, 97.5% values are shown.

^†^The data did not support between-patient variability of the windowed parameters for HBV patients.

#### Case control analysis

To test prospectively the algorithm developed above, we designed a case control study using the 32 cases who triggered in our dynamic Bayesian analysis, 29 of whom were HCV patients. For each trigger-case and trigger-year, we selected five matched controls from the ‘*HCC surveillance cohort*’–see **Patients and Methods** section for matching details. One control had HCC diagnosed in December 2008 (and therefore should have been excluded, but was not due to lack of clinical information prior to the detailed look-back). We have retained this control as an HCC-case, using OLT-date in 2009 as the event-month.

In 10 HCV case-control sets, HCC developed in 11 patients (Table 4); 6 of the 29 cases which triggered the algorithm developed HCC. In five sets, the trigger-case but none of the five controls developed HCC. The probability of this most-extreme out-turn is [1/6]^5^ or 1.286 * 10^−4^. In four sets, one of the five controls was subsequently diagnosed with HCC but not the trigger-case. The probability of this, or a more extreme, out-turn is [5/6]^4^ + [1/6]^4^ or 0.483. In the final set, there were two HCC diagnoses, the HCV trigger-case and one of five controls. The probability of this, the most extreme out-turn for two HCC diagnoses, is 5/15 or 0.333. In summary, had the chance of HCC diagnosis during 2009–2014 been identical for HCV trigger-cases and controls, the probability of outcomes as extreme as we have observed when multiplying across our 10 informative case-control sets, is extremely low: 2.07 * 10^−5^.

**Table 4:** Case-controls study assessing the performance of our dynamic Bayesian algorithm for identifying HCV cases at higher risk of HCC-diagnosis during 2009–2014.

Case ID	Diagnosis, Gender, Age @2009	1^st^ trigger year	Death Month/yr	HCC Month/yr	OLT Month/yr	TACE Month/yr	RFA Month/yr	Person-months
**11**	**HCV, m, 56**	**2007**	Jul-13	Jun-11			Sep-11	**30**
5controls			Jun-11^a^	*	*			7+246 = 253
				†Dec-08^b^	†Jul-09^b^			
**39**	**HCV, m, 56**	**2006**	*	Jun-11	*	*	*	**30**
5controls								360
**241**	**HCV, f, 69**	**2010**		Dec-14				**60**
5controls			Mar-12					267
**326**	**HCV, f, 66**	**2002**	*	*	*	*	*	**72**
5controls			Mar-12^a^	Dec-14^b^				327
**474**	**HCV, m, 62**	**2008**	*	*	*	*	*	**72**
5controls			Jan-10					301
**487**	**HCV, f, 62**	**2006**		Mar-14^a^	Jul-14^a^	Jul-14^a^		**63**
5controls			Mar-12					327
**617**	**HCV, m, 59**	**2007**	*	*	*	*	*	**72**
5controls			Oct-09					298
**632**	**HCV, m, 58**	**2011**	*	*	*	*	*	**48**
5controls			Apr-14^a^	Apr-13^a^				220
**646**	**HCV, m, 58**	**2008**	*	*	*	*	*	**72**
5controls			Oct-09^a^					279
			13^b^	May-13^b^				
**754**	**HCV, m, 56**	**2010**	Apr-14^a^	Apr-13^a^				**40**
5controls								300
**809**	**HCV, m, 55**	**2007**	Apr-10					**16**
5controls								360
**812**	**HCV, f, 55**	**2003**	*	*	*	*	*	**72**
5controls								360
**818**	**HCV, f, 55**	**2009**	*	*	*	*	*	**72**
5controls			Jul-12^a^		Aug-10^b^			308
**827**	**HCV, m, 55**	**2005**	*	*	*	*	*	**72**
5controls				Jun-13^a^	Feb-14^a^	Dec-13^a^		342
**840**	**HCV, m, 54**	**2010**		Apr-14^a^		Jun-14^a^		**52**
5controls								300
**848**	**HCV, f, 54**	**2009**	*	*	*	*	*	**72**
5controls								360
**859**	**HCV, m, 54**	**2009**	*	*	*	*	*	**72**
5controls								360
**926**	**HCV, m, 53**	**2006**	*	*	*	*	*	**72**
5controls								360
**936**	**HCV, m, 52**	**2002**	Jun-11					**30**
5controls								360
**938**	**HCV, m, 52**	**2006**	*	*	*	*	*	**72**
5controls			Dec-13					348
**943**	**HCV, f, 52**	**2009**	*	*	*	*	*	**72**
5controls			Jan-14					349
**1008**	**HCV, m, 51**	**2009**	*	*	*	*	*	**72**
5controls					Apr-11			316
**1156**	**HCV, m, 47**	**2009**	*	*	*	*	*	**72**
5controls			Dec-13^a^		Apr-13^a^			340
**1164**	**HCV, f, 46**	**2004**	Sep-11					**33**
5controls			Feb-11					314
**1223**	**HCV, m, 45**	**2009**	Oct-14					**70**
5controls			Feb-12					326
**1233**	**HCV, m, 44**	**2006**	*	*	*	*	*	**72**
5controls								360
**1359**	**HCV, m, 40**	**2007**	May-10					**17**
5controls			Apr-13^a^					338
			Oct-14^b^					
**1378**	**HCV, f, 39**	**2010**	*	*	*	*	*	**60**
5controls			May-11^a^					247
			Feb-14^b^					
**1403**	**HCV, m, 37**	**2010**			Dec-13			**60**
5controls			Dec-09^a^					12+226 = 238
			Oct-13^b^					
**cases**		**29**	**7**	**6**	**2**	**2**	**1**	**1689**
**controls**		**145**	**21**	**5**	**5**	**1**	**0**	**9218**
**Non HCV cases**								
**39**	**NFALD,m,79**	**2005**	2012					
2controls			2010^a^					
			2013^b^					
**890**	**ALD, f, 53**	**2011**	2011					Not analysed
4controls			2014					
**1148**	**ALD, m, 47**	**2011**			Jun-13			
5controls			2011^a^		Jul-10^d^			
			2012^b^					
			2013^c^					

Individual cases triggering the defined algorithm are presented by their identifier (ID) within the *AFP surveillance cohort* together with basic demographic data used to match 5 controls. Columns highlight cases of death, HCC diagnosis, OLT, and either TACE or RFA non-surgical management. Person-months denotes the follow-up period. One control for case 818 had HCC based upon a single CT, but no HCC was detected upon repeat imaging (with MRI and CT) prior to OLT without specific anti-HCC therapy, nor at transplant explant examination seven months later. This case was therefore not listed as HCC in the database nor considered as *bona fide* HCC in the case-control assessment.

^†^ Denotes a patient who, upon review, was diagnosed with HCC prior to the point of patient inclusion of 1/1/2009 but is analysed by OLT-date.

*denotes absence of events.

Superscript letters are used to differentiate separate individuals in the control groups.

Finally, our 29 HCV trigger-cases experienced six HCC diagnoses in 1,689 person-months at-risk during 2009–2014, an HCV trigger-case diagnosis-rate of 4.3 HCCs diagnosed per 100 person-years at-risk (95% CI: 1.56 to 9.28). For the 145 HCV controls, there were five HCC diagnoses in 9,218 person-months at-risk during 2009–2014, an HCV controls’ diagnosis-rate of 0.65 HCCs per 100 person-years at-risk (95% CI: 0.21 to 1.52), which is significantly lower as clearly indicated by non-overlapping Poisson-based 95% confidence intervals.

Retrospective review of the case histories of the six HCV cases who triggered an ‘at risk alert’ by the algorithm and went on to develop HCC (Table 4) revealed that, in two of the six cases a HCC was detected by a lesion on US leading to diagnosis on initial cross sectional imaging within 3 months. Another one of the six cases had a prior lesion on US which was subsequently followed up by cross sectional imaging leading to HCC diagnosis 13 months subsequently. Hence, in half of these six cases AFP did not significantly affect HCC detection. In only one case did the rising AFP lead to a direct change of patient surveillance management. Here, a clinician detected the rising AFP (without the help of the algorithm) and converted surveillance to regular cross sectional imaging with ongoing AFP monitoring. The AFP then continued to rise and HCC was diagnosed 36 months later (AFP—355 at diagnosis) and treated with local regional therapy. The patient was still alive at census. In the last two cases in which the algorithm detected ‘high risk’ HCC behaviour during screening prior to HCC development, the AFP did not change clinical management and the HCC was detected incidentally by cross sectional imaging performed for decompensated liver disease (jaundice and ascites). In both cases an US was performed within 6 months of HCC diagnosis and detected no clinically significant lesion. Of these two patients, one was dead within a year and received no specific HCC-therapy; the other received a liver transplant 4 months after diagnosis and following loco-regional HCC therapy and was alive at census.

## Discussion

The objective of this study was to assess in patients within an HCC surveillance programme the potential of serial AFP analysis to improve detection of treatable HCC specifically where US shows no lesion. Here we report the local experience of using AFP over a five year period in a well-defined and followed up loco-regional series of patients undergoing AFP surveillance and/or management of HCC. In our hands, the use of serum AFP in HCC surveillance has facilitated the early diagnosis of HCC in a large proportion of the patients undergoing HCC surveillance in whom the HCC was otherwise not detected by US alone. Despite this group’s elevated AFP levels at HCC diagnosis, potentially curative therapy was offered in the majority of instances, including offers of surgical therapy (resection and or liver transplant). Importantly, the AFP-detected HCC cases were not disadvantaged in terms of survival. We therefore believe our data provide good evidence for the use of AFP in HCC surveillance programmes.

We therefore set out to develop mathematical model to aid healthcare workers in discriminating between benign variability in AFP, and variations which may reflect the development of an underlying HCC. Such a model should provide a standardised accurate and validated interpretation of AFP results and ideally have the potential to be optimised iteratively when further data is available.

In this proof of principle study we show that an automated algorithm for monitoring dynamic changes in AFP is able to identify patients at high risk for HCC development. The cases analysed in this study had principally HCV related liver disease. This algorithm has the advantage that it may be further refined by the inclusion of larger data sets, including those in which other aetiologies predominate. The dynamic Bayesian approach has the potential to offer an automated and validated interpretation of an individual’s screening AFP results in the context of other in the same aetiological group. It takes into account the individual’s AFP history as well as his/her most recent six AFPs and other information: specifically, aetiology of liver disease. Automating such an analysis and adding a user friendly interface would provide immediate data interpretation following biochemical analysis and unambiguous interpretation of results by specialists and non-specialists alike. Lack of consensus on how to interpret results from AFP screening is a significant barrier to performing optimal HCC surveillance even in westernised healthcare systems and such a system could therefore provide a much needed improvement.

Currently this study has been restricted to a subset of the total cohort of patients undergoing HCC surveillance. A clear limitation to this proof of concept study is that is has not been validated, to date, in an independent external cohort. Currently we have examined the utility of this approach for four main aetiologies and only for screened patients with at least six AFP measurements and evidence of linearity: 672 out of a total of 1148 patients (59%) whose aetiology was ALD, HCV, NAFLD or HBV. However this corresponds to 83% of the 814 with at least six AFP measurements, our minimum threshold for establishing patient-specific log-AFP trajectories. In total, by addition of 22 Lothian patients with these aetiologies whose HCC was diagnosed early and had at least six AFPs, 694 patients were included in the derivation of this algorithm and we would plan to extend this in future work.

The restriction to early-diagnosed HCC cases was quite limiting in terms of the number of patients whose log-AFP screening profile could be learned from and so future work will: i) increase the pool of early-diagnosed HCC cases by which to ‘train’ the Bayesian analysis in Lothian; ii) validate the Lothian algorithm for external cohorts; iii) try reducing the entry-threshold from six AFPs, as Bayesian learning between wave 1 and 48 was modest; and iv) test performance of the algorithm when a training set admits HCC cases from the four main aetiologies whose diagnosis was not made early enough for management with curative intent to have been offered. We include this fourth proposition because, although we had hoped that trigger-cases would be imminently an early-HCC-diagnosis, what we have shown is that the algorithm identifies trigger-cases who demonstrated significantly higher risk of HCC diagnosis during 2009–2014. This being so, cases in whom HCC was diagnosed late might have triggered the Bayesian algorithm earlier. Fifth, in line with previous calls to do so [22], our Bayesian algorithm could be adapted to include other markers such as ALT and other covariates than aetiology, such as sex or ethnicity which has been shown to influence AFP values [13, 18, 19]. Given previous reports of improved AFP levels in patients achieving sustained virological response (SVR) to anti-Hepatitis C therapy [38], SVR could also be introduced to patient-specific algorithms in the future.

Validation on an external cohort is particularly relevant when considering the potential impact of other factors such as ethnicity or SVR and because variation in aetiological incidence may account for the relatively low HCC incidence in our cohort (below 2% per year). Further work is also required for the cohort of patients whose log-AFPs do not conform to linearity.

It is clear that AFP will not provide a faultless screening mechanism independent of, or combined with, US based imaging. Consistent with previous reports [39], many patients in our cohort remain with low AFP levels even following extensive HCC development. The secretion of AFP may in itself identify a separate molecular signature [40]. Alternatively, extreme elevations of AFP appear to be present in a group in which HCC surveillance is futile due to poor outcome [33]. A frequent finding in Lothian was that a rising AFP will trigger CT/MRI which detects an indeterminate lesion, not fulfilling HCC diagnostic criteria. Ongoing follow-up in such cases remains a dilemma. Consistent with previous reports [41], such indeterminate lesions in our study often develop HCC defining criteria over a further follow up period. In our study, we recognised a subgroup of such patients in whom further elevations of AFP were able to provoke earlier reimaging leading to earlier diagnosis.

As reflection of real world practice, it is noteworthy how many patients undergoing screening had their HCC detected by other means including clinical trials and imaging performed for other indications outwith the surveillance programme (see Fig 1c). Similarly despite an intention to maintain 6-monthly surveillance testing patients did not always have these tests arranged or performed. This variability in recall and attendance is another feature which often fails in surveillance programmes and is a further target for improving surveillance programmes [42].

Numerous related studies have similarly reported on the interpretation of dynamic AFP changes for specific patients. These studies have similarly focused upon chronic viral hepatitis [15, 17, 23–25, 27, 43] and have typically used variable cut off values for AFP and/or rates of AFP elevation—either as multiples of a baseline or over a set time interval both of which produce potential difficulties when interpreting data over variable and often unpredictable time intervals. Our well characterized cohort is comparable in size to the largest of these previous studies [24], the dynamic Bayesian algorithm learns from other patients within the same aetiology, and is potentially more flexible to future refinement than those described previously, and is not constrained by fixed time intervals. Furthermore our approach offers the advantage of analysis of AFP data samples collected retrospectively, but analysed as if they were occurring in real time. The advantage of our approach in creating an automated interpretation is that risk stratification can be automated and delivered direct to the clinician. The current methodology, which tested both the rate of AFP elevation and its absolute value relative to that individual’s history, provides an important proof of principle for the application of Bayesian analysis in the development of a sophisticated analytical tool to provide much needed addition to ultrasound in the surveillance of HCC.

Should subsequent validation in larger independent cohorts also support the utility of an algorithmic approach to AFP monitoring in HCC, we would propose that clinicians should convert US based surveillance to cross sectional imaging based surveillance in patients identified as high risk by the algorithm for a time dependent on ongoing AFP activity and the presence or absence of any indeterminate lesions seen on imaging.

## Supporting Information

S1 Fig Individual patient’s longitudinal AFP history over time is shown for the 32 cases which triggered the AFP analysis algorithm. (TIF)

S1 Supporting Methods (DOCX)

S1 Table Characteristics of HCCs at the time of diagnosis in the *HCC case series*. (DOCX)

S2 Table Details of the clinical pathway for HCC detection in the 28 patients where AFP altered management leading to HCC detection in whom a recent (≤6 months) US was not performed. (DOCX)

S3 Table Checks prior to definitive Bayesian analysis. (DOCX)

S4 Table Lothian sub-cohort of patients with at least six AFPs: support for linearity based on all AFPs or windowed-in on the most recent six AFPs. (DOCX)
