# Supplementary figures and images for: Alpha-Fetoprotein Detection of Hepatocellular Carcinoma Leads to a Standardized Analysis of Dynamic AFP to Improve Screening Based Detection
Source: PLoS One. 2016 Jun 16;11(6):e0156801. doi: 10.1371/journal.pone.0156801 (PMC4911090; doi:10.1371/journal.pone.0156801)

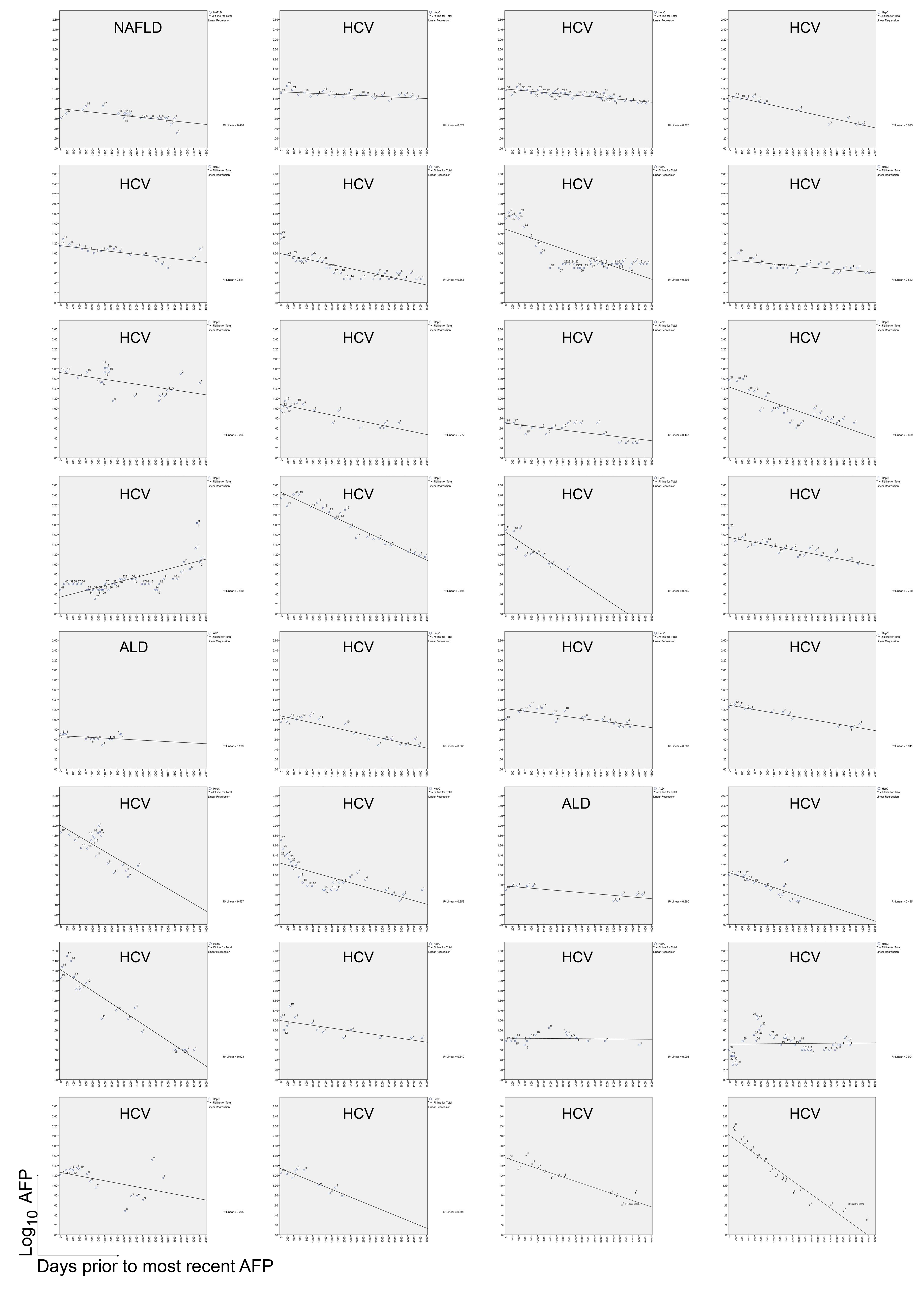

Supplement: S1 Fig — (TIF) [file pone.0156801.s001.tif]
