# Supplementary material for: Alpha-Fetoprotein Detection of Hepatocellular Carcinoma Leads to a Standardized Analysis of Dynamic AFP to Improve Screening Based Detection
Source: PLoS One. 2016 Jun 16;11(6):e0156801. doi: 10.1371/journal.pone.0156801 (PMC4911090; doi:10.1371/journal.pone.0156801)
Supplement: S1 Supporting Methods — (DOCX) [file pone.0156801.s002.docx]

# **Supporting Methods**

## **Logarithmic transformation of AFP**

The standard deviation for AFP increases as mean AFP does, and so key analyses were performed after log10-transformation. On the scale of log_10_-AFP (hereafter log-AFP), standard deviation was typically around 0.1, and increased only slightly as mean log-AFP did, demonstrating that the transformation was adequate. There was no evidence that mean-log-AFP varied systematically by age-at-screen. There was also no evidence that the length of inter-screen intervals was informative about AFP-levels.

## **Bayesian analysis plan**

Initial insights: The Bayesian analysis initially contrasted linearity for the four major diagnostic groups by analysing only those patients for whom there was adequate support for linearity either on the basis of all log-AFPs or of the most recent six log-AFPs. This initial analysis, which fitted an indicator for early-diagnosed HCC cases, confirmed that the log-AFP intercept was highest and gradient most strongly negative for early-diagnosed HCC cases; with the HCV sub-cohort next in line.

The initial insights Bayesian set-up allowed estimation of parameters (such as aetiological-mean intercepts) when based on all screened AFPs or when windowed-in on the most recent six. Aetiology-specific standard deviations measure how variable patient-specific estimates are about their aetiological-means – whether in terms of intercept or gradient.

## **Formal Bayesian analysis in static and dynamic modes:**

Ultimately, we needed to apply our Bayesian analysis dynamically once a patient had achieved six or more AFPs and sufficient evidence of linearity. Early-diagnosed HCC cases could, and should, now sit within their aetiology, rather than have a separate indicator – as at the phase of initial insights, which had served its purpose.

In dynamic mode, the first stage of the analysis is to estimate support for linearity as each screen is added; only those patients with adequate-to-date-support for linearity are admitted into the dynamic analysis. The training data-set, thus dynamically created, was initially analysed in static mode to determine trigger-regions, defined by patients’ estimated log-AFP intercept and gradient, in which most of the early-diagnosed HCC cases were located. The training data-set was then analysed dynamically to establish the likely recall/re-screening burden occasioned by possibly-repeated triggering as a patient’s data accumulated.

## **Bayesian analysis: static mode:**

Let m_i_ denote the total number of observations available for individual i. All data were included in this analysis: observations 1 to m_i_ (for each individual i) for `full-data' analysis; and observations m_i_ - 5 to m_i_ for `windowed' analysis (see below for definition of m_i_). Estimated patient-specific intercepts were plotted against the corresponding gradients, with different symbols for the HCC cases. The Bayesian analysis in static mode was used to identify areas of parameter-space that might indicate emerging HCC cases – termed ‘trigger zones’ identified by the area to the upper left of the purple lines in **Figs 3b** and **c**.

## **Bayesian analysis: waves of analysis in dynamic mode**

Let p_i_ denote the "point of entry" (into our prospective study) for individual i. Traversing his/her data chronologically, “point of entry” is defined as the observation number at which his/her AFPs first provide an R^2^ greater than 0.3, either over the most recent six AFPs or over all AFPs up to and including the most recent.

Our dynamic analysis comprises 48 waves, indexed by w. For wave 1, our data-set comprises all AFPs up to (and including) the point of entry for each individual. We imagine that all individuals are `synchronised' at this point and that subsequent AFP measurements also arrive in synchronised fashion. The data-set grows in each subsequent wave such that a single additional AFP reading is included for each individual, if available.

For individual i in wave w, let m_i_ = min (n_i_, p_i_ + w - 1). (In our dataset, the maximum number of additional AFPs after point of entry is 47, hence the 48 waves.) The `full-data' analysis includes observations 1 to m_i_ for each individual, whereas our `windowed' analysis includes observations m_i_ - 5 to m_i_. Note that wave 48 corresponds to the static analysis described above.

For each individual, and within each wave, the *posterior* probability of his/her regression parameters lying within each of the trigger-zones identified above was estimated. If this posterior probability is greater than a specific threshold (chosen as 0.67 for case/control assessment) then a "trigger" is said to have occurred for that individual.

We studied the pattern of triggers over the 48 waves for each patient, to establish whether we could identify any who might soon develop HCC (including the early-diagnosed HCC cases in the training data). These trigger-cases and matched controls were also used to inform a basic cost-effectiveness analysis (not reported here).

In each wave of the Bayesian analysis, the following hierarchical model was fitted to log-AFP values, where $y_{ij}$represents log-AFP for patient *i* at measurement time *j*. The covariate $z_{ij}$ represents number of days since last screening. Intercept and gradient are denoted by $\alpha_{i}^{k}$and $\beta_{i}^{k}$ respectively, and allowed to differ across the four aetiologies ALD, HCV, NAFLD and HBV, which are distinguished by indicators $x_{i1}$ to$x_{i4}$respectively where indicator $x_{i1}$ takes the value 1 if patient *i*’s aetiology is ALD and zero otherwise; indicator$x_{i4}$takes the value 1 if patient *i*’s aetiology is HBV and zero otherwise. Within each aetiology (denoted by k), intercepts and gradients differ randomly across patients.

**Table 3** reports means and variances of the intercepts and gradients, for each of the four aetiologies. The model parameters are summarised by posterior medians and 2.5% and 97.5% percentiles from the posterior distributions. Results are reported for the full-data and windowed Bayesian analyses carried out in static mode (wave 48) and for wave 1 in dynamic mode.
