# Supplementary material for: Alpha-Fetoprotein Detection of Hepatocellular Carcinoma Leads to a Standardized Analysis of Dynamic AFP to Improve Screening Based Detection
Source: PLoS One. 2016 Jun 16;11(6):e0156801. doi: 10.1371/journal.pone.0156801 (PMC4911090; doi:10.1371/journal.pone.0156801)
Supplement: S1 Table — (DOCX) [file pone.0156801.s003.docx]

## **S1 Table: Characteristics of HCCs at the time of diagnosis in the HCC case series.**

^*^2 patients did not have AFP level in 1 year prior to diagnosis, ^‡^Single HCC ≤50mm, or upto 5 HCC maximum diameter 30mm, Potentially curative therapy includes resection, RFA, liver transplant, ^†^denotes part of multiple therapy, # includes one patient active on waiting list, ^!^Patients listed for OLT but delisted subsequently
