# Supplementary material for: Alpha-Fetoprotein Detection of Hepatocellular Carcinoma Leads to a Standardized Analysis of Dynamic AFP to Improve Screening Based Detection
Source: PLoS One. 2016 Jun 16;11(6):e0156801. doi: 10.1371/journal.pone.0156801 (PMC4911090; doi:10.1371/journal.pone.0156801)
Supplement: S2 Table — (DOCX) [file pone.0156801.s004.docx]

**S2 Table: Details of the clinical pathway for HCC detection in the 28 patients where AFP altered management leading to HCC detection in whom a recent (≤6 months) US was not performed.**

.
